# Use of clinical pharmacy services by American Indians and Alaska Native adults with cardiovascular disease

**DOI:** 10.1002/jac5.1651

**Published:** 2022-05-25

**Authors:** Joan O'Connell, Laura Grau, Spero M. Manson, Anne Marie Bott, Kyle Sheffer, Randy Steers, Luohua Jiang

**Affiliations:** ^1^ Present address: Centers for American Indian and Alaska Native Health, Colorado School of Public Health University of Colorado Aurora Colorado USA; ^2^ Present address: Department of Biostatistics, Colorado School of Public Health University of Colorado Aurora Colorado USA; ^3^ Alaska Native Medical Center Anchorage Alaska USA; ^4^ Santa Fe Indian Health Center Santa Fe New Mexico USA; ^5^ Chickasaw Nation Medical Center Ada Oklahoma USA; ^6^ Department of Epidemiology and Biostatistics University of California Irvine California USA

**Keywords:** cardiovascular disease, American Indian, health services research, medication management, outcomes, population health, social determinants of health

## Abstract

**Introduction:**

The Indian Health Service (IHS) and Tribal health programs provide clinical pharmacy services to improve health outcomes among American Indian and Alaska Native (AI/AN) adults with cardiovascular disease (CVD).

**Objectives:**

The study's primary objective was to describe characteristics, including social determinants of health (SDOH), associated with clinical pharmacy utilization by AI/ANs with CVD who accessed IHS/Tribal services. A secondary objective assessed changes in systolic blood pressure (SBP) associated with such utilization.

**Methods:**

Analysis included IHS data for 9844 adults aged 18 and older with CVD who lived in 5 locations. Multivariable logistic regression was used to examine patient characteristics (eg, age, sex, health status, SDOH) associated with clinical pharmacy utilization in fiscal year (FY) 2012. A propensity score model was employed to estimate the association of elevated SBP in FY2013 with FY2012 clinical pharmacy utilization.

**Results:**

Nearly 15% of adults with CVD used clinical pharmacy services. Among adults with CVD, the odds of clinical pharmacy use were higher among adults diagnosed with congestive heart failure (adjusted odds ratio [OR] = 1.22; 95% CI:1.01‐1.47), other types of heart disease not including ischemia (OR = 1.40; 95% CI: 1.18‐1.65), and vascular disease (OR = 1.23; 95% CI: 1.04‐1.46), compared to adults without these conditions. Diabetes (OR = 4.05, 95% CI: 3.29‐5.00) and anticoagulation medication use (OR = 20.88, 95% CI: 16.76‐20.61) were associated with substantially higher odds of clinical pharmacy utilization. Medicaid coverage (OR = 0.72; 95% CI: 0.56‐0.93) and longer travel times to services (OR = 0.87; 95% CI: 0.83‐0.92) were each associated with lower odds. FY2012 clinical pharmacy users had lower odds of elevated SBP (OR = 0.71 95% CI: 0.58‐0.87) in FY2013 than nonusers.

**Conclusion:**

In addition to health status, SDOH (eg, Medicaid coverage, longer travel times) influenced clinical pharmacy utilization. Understanding characteristics associated with clinical pharmacy utilization may assist IHS/Tribal health programs in efforts to support optimization of these services.

## 1 INTRODUCTION

American Indian and Alaska Native (AI/AN) peoples experience significant morbidity and mortality due to chronic disease,^1^, ^2^, ^3^, ^4^, ^5^, ^6^, ^7^ most notably high rates of heart disease, stroke, and diabetes.^5^, ^6^, ^7^ AI/AN all‐cause mortality rate was found to be 46% higher than that of non‐Hispanic Whites in geographic locations served by the Indian Health Service (IHS).^1^ This difference was largely attributable to disparities in mortality associated with heart disease, stroke, diabetes, and kidney disease.^1^ Structural racism combined with continued disparities in determinants of health^8^, ^9^, ^10^, ^11^ and existing AI/AN cardiovascular disease (CVD) risk factors^12^ contribute to higher rates of premature death due to heart disease, stroke, and diabetes among AI/ANs.^2^, ^3^, ^4^

Innovative service delivery models are needed to address CVD risk factors among AI/ANs, particularly in rural areas with low household incomes.^8^, ^13^, ^14^ IHS provides health care funding for approximately 2.6 million AI/ANs who obtain services through facilities operated by IHS, Tribes, and urban Indian organizations (I/T/Us).^15^ Clinical pharmacy services are an integral component of the medical home model used by I/T/Us.^16^, ^17^, ^18^, ^19^ As the number of AI/ANs aged 65 and older increases—expected to more than triple by 2050^20^—the need for chronic disease services also increases. A shortage of primary care providers (PCPs) compounds this challenge.^8^, ^19^, ^21^, ^22^ Increasingly, clinical pharmacists aid in meeting AI/AN patients' needs for chronic disease services.

The 2011 U.S. Surgeon General report *Improving Patient and Health System Outcomes through Advanced Pharmacy Practice* summarized evidence of positive patient and health system outcomes associated with the provision of direct patient care by pharmacist providers.^16^ Within the IHS service delivery system, pharmacist providers may act as consultants to physicians and mid‐level PCPs or as a PCP.^16^, ^19^ As consultants, pharmacists provide medication recommendations optimizing patient care without prescriptive authority. Alternatively, many clinical pharmacists have authority to order and interpret laboratory tests, perform physical assessments, and have prescriptive authority. The *U.S. Public Health Service National Clinical Pharmacy Specialist* (NCPS) *Committee* establishes national standards for clinical pharmacy protocols and collaborative practice agreements to support I/T/Us in the provision of quality clinical pharmacy services.^23^

A growing body of evidence demonstrates the value of clinical pharmacy services, which have evolved from chronic disease state management to comprehensive medication management (CMM).^24^ Clinical pharmacy services improve health outcomes and reduce risks of adverse drug events and medication errors,^16^, ^25^, ^26^, ^27^, ^28^, ^29^, ^30^, ^31^, ^32^, ^33^, ^34^ particularly among patients with multiple chronic diseases or who use high‐risk medications. Among patients with CVD, clinical pharmacy services have been associated with improvements in patient medication adherence and reductions in CVD risk factors (eg, high systolic blood pressure [SBP], high cholesterol, and smoking).^28^, ^29^, ^30^, ^31^, ^32^

In 2019, the NCPS Committee reported the impact of NCPS‐certified pharmacist providers in federal agencies, including IHS.^19^ Patients with hypertension were able to reduce both systolic and diastolic blood pressure values to levels within standard goals. More than 22% of patients using clinical pharmacy tobacco cessation services ended tobacco use after 6 months of initiating services. A 2015 report on the Yakama Indian Health Center documented that patients with diabetes who utilized clinical pharmacy services were more likely to have their glucose, blood pressure, and cholesterol under control than patients with diabetes who did not.^18^

To date, no reports have characterized clinical pharmacy utilization among AI/ANs with CVD, using data for both users and nonusers. With a high burden of CVD among AI/ANs and the increasing numbers of AI/AN older adults with multiple chronic conditions,^20^ it is critical to identify potential barriers and benefits of clinical pharmacy services. The primary goal of this study was to use existing IHS data to examine characteristics associated with clinical pharmacy service utilization among AI/ANs with CVD. A secondary goal assessed SBP rates associated with its use.

## 2 METHODS

### 2.1 Data source

Our study drew upon data from the IHS *Improving Health Care Delivery Data Project* (IHS Data Project). The IHS Data Project's data infrastructure includes information for a purposeful sample of AI/ANs who lived in 15 IHS Service Units (hereafter referred to as geographic areas) located throughout the United States. Sources of IHS electronic data include the National Data Warehouse (NDW) and Purchased and Referred Care (PRC) data. The IHS Data Project population is comparable to the national IHS service population in terms of age and gender.^35^ This study included data from five geographic areas where 5% or more adults with CVD had one or more clinical pharmacy visits. Among the 10 Service Units excluded from this analysis, 3 did not provide clinical pharmacy services during the study time period; 1 had a data quality issue; and 6 had low utilization where, in general, clinical pharmacy services were being initiated or provided on a very limited basis.

Project personnel partnered with IHS and the Tribal organizations that participate in the IHS Data Project.^35^ Partnership included meetings with the project's Collaborative Network (composed of three advisory committees: Steering, Project Site, and Patient), travel to project sites, and a process to obtain approvals from the IHS Institutional Review Board (IRB), Tribal IRBs, and Tribal Councils and Authorities in addition to the university's IRB.

### 2.2 Study population

A total of 11 854 AI/AN adults with CVD used I/T services during each of the fiscal years (FY) FY2011‐FY2013 (ie, October 1, 2010‐September 30, 2013) and lived within the five geographic areas. Adults with missing county or community data (n = 390) or who were diagnosed with end‐stage renal disease (ESRD), transplants, or malignant cancer (n = 1618), due to their complex treatment needs, were excluded from the analysis. The study population included 9844 adults with CVD.

We first examined associations between age, health coverage, health status, SBP, low‐density lipoprotein (LDL) cholesterol, hemoglobin A1c (HbA1c), and county and community social determinants of health (SDOH) with FY2012 clinical pharmacy utilization. Second, we assessed the relationship between FY2012 clinical pharmacy use with SBP during the following year (FY2013).

### 2.3 Measures

#### 2.3.1 Demographic and health coverage

NDW FY2011 data provided information on age, sex, location, and health coverage (ie, Medicare, Medicaid, private insurance, no coverage in addition to access to IHS services).

#### 2.3.2 Health status

We employed international *Classification of Diseases ninth Revision Clinical Modification* (ICD‐9) diagnostic codes, recorded in the NDW and PRC inpatient and outpatient service use records, supplemented by glucose values and medication data, to create dichotomous FY2011 chronic condition variables that indicate an adult had, or did not have, a chronic disease. Sightlines DxCG Risk Solutions^36^ was used to identify adults with CVD, hypertension, mental health and substance use disorders, and other types of chronic disease except for diabetes and ESRD for which we used project specific algorithms, developed from nationally recognized references.^37^, ^38^

Based on IHS and national guidelines at the time of the study, we defined FY2011 and FY2013 elevated SBP as SBP ≥140 mm Hg, FY2011 elevated LDL cholesterol as values ≥100 mg/dL, and FY2011 elevated hemoglobin A1c as HbA1c ≥8%.^39^, ^40^ For these measures, missing data was either due to the patient not having the procedure/test or the value not being recorded.

#### 2.3.3 Health services utilization

We defined an FY2011 4‐level outpatient medication use variable based on quartiles (Q) of dispensed medications: Q1 (≤27 medications), Q2 (28‐61), Q3 (62‐100), and Q4 (>100). Adults who received any anticoagulation medications in FY2011 were classified as anticoagulant users. One or more clinical pharmacy visits in FY2012 classified adults as users.

#### 2.3.4 Social determinants of health

Pursuant to the Healthy People 2030 SDOH framework,^41^ we organized 4 SDOH measures as: *health care access and quality measures*: individual health coverage and community travel times; *education access and quality measure*: county‐level educational attainment; and *economic stability measure*: county‐level household income. Three SDOH measures, derived from other sources, were linked to each adult based on where they lived in FY2012. County‐level household income and educational attainment data were obtained from 2010 to 2014 American Community Survey data for AI/ANs who reported access to IHS services.^42^ We defined lower and higher poverty counties and lower and higher educational attainment counties using the medians among all the counties included in this study for the percentage of AI/AN households with incomes below 139% of the federal poverty level (FPL, median = 35.4%) and the percentage of AI/ANs aged 25 with less than a high school degree (median = 43.2%). Household income below 139% of the FPL is used by many states to determine one type of Medicaid eligibility. A community's average patient travel (drive) time to clinical pharmacy services in FY2012 was estimated from a central location in their community to an I/T facility that provided clinical pharmacy services, using geocodes and OpenStreetMaps.^43^, ^44^

### 2.4 Analysis

SAS 9.4 was used to analyze the data.^45^ We report findings for adults with CVD and for 2 subgroups: adults with both CVD and diabetes and those with CVD but not diabetes. Characteristics were compared between clinical pharmacy users and nonusers, using Chi‐Squared tests, or two sample t‐tests at the individual level, or univariate logistic regressions with random intercepts at site, county, or community level. Multivariable logistic regression models were utilized to predict service use, including site fixed effects and random intercepts for counties and communities. Adjusted odds ratios (ORs) are presented with 95% confidence intervals (CI).

For the secondary study goal, we employed a propensity score matched conditional logistic regression and multivariable logistic regression to estimate the association between clinical pharmacy use in FY2012 with elevated SBP in FY2013 among adults with CVD.^46^ This statistical approach was selected to control for potential bias due to patient self‐selection into use or nonuse of clinical pharmacy services in this observational study. We conducted a sensitivity analysis by estimating the same relationship using an alternative statistical method developed to control for selection bias, the Disease Risk Score model.^47^ This analysis only included data for 4 of the 5 geographic areas due to SBP data quality issues.

## 3 RESULTS

### 3.1 Baseline characteristics

Over 40% of adults with CVD were age 65 years and older; 50.3% were female. Approximately 10% had Medicaid coverage, 51.7% had Medicare coverage, and one‐fourth had private insurance. Nearly 30% reported no health coverage other than access to IHS services (see Table S1).

### 3.2 Use of clinical pharmacy services

In FY2012, 1462 (14.9%) adults with CVD used clinical pharmacy services (Table 1). Users averaged 5.3 clinical pharmacy visits during FY2012 (Table S2). Use increased with age; males had higher use than females. Adults with Medicare coverage had significantly higher use than adults without Medicare coverage, while adults who reported no health coverage other than access to IHS services had significantly lower use than adults with health coverage.

**TABLE 1:** Fiscal year 2011 characteristics^a^ of American Indian and Alaska Native adults with cardiovascular disease (CVD) by health status and use of clinical pharmacy (CP) services in fiscal year 2012. Five geographic locations

	Health status											
	CVD				CVD and diabetes				CVD absent diabetes			
	CP nonuser	CP user	*P*‐value	CP use rate	CP nonuser	CP user	*P*‐value	CP use rate	CP nonuser	CP user	*P*‐value	CP use rate
Characteristics	# (%)	# (%)		%	# (%)	# (%)		%	# (%)	# (%)		%
All adults	8382 (100.0)	1462 (100.0)		14.9	3528 (100.0)	1154 (100.0)		24.6	4854 (100.0)	308 (100.0)		6.0
*Demographic*												
Age (years)												
18‐35	541 (6.5)	25 (1.7)	<.001	4.4	53 (1.5)	15 (1.3)	.002	22.1	488 (10.0)	10 (3.3)	<.001	2.0
35‐45	685 (8.2)	68 (4.7)		9.0	181 (5.1)	45 (3.9)		19.9	504 (10.4)	23 (7.5)		4.4
45‐55	1610 (19.2)	212 (14.5)		11.6	633 (17.9)	164 (14.2)		20.6	977 (20.1)	48 (15.6)		4.7
55‐65	2, 238 (26.7)	402 (27.5)		15.2	1079 (30.6)	341 (29.6)		24.0	1159 (23.9)	61 (19.8)		5.0
65+	3308 (39.5)	755 (51.6)		18.6	1582 (44.8)	589 (51.0)		27.1	1726 (35.6)	166 (53.9)		8.8
Sex			.001				.888				<.001	
Male	4105 (49.0)	783 (53.6)		16.0	1832 (51.9)	602 (52.2)		24.7	2273 (46.8)	181 (58.8)		7.4
Female	4277 (51.0)	679 (46.4)		13.7	1696 (48.1)	552 (47.8)		24.6	2581 (53.2)	127 (41.2)		4.7
*Health coverage*												
Medicaid	814 (9.7)	127 (8.7)	.219	13.5	334 (9.5)	100 (8.7)	.415	23.0	480 (9.9)	27 (8.8)	.521	5.3
Medicare	4169 (49.7)	918 (62.8)	<.001	18.0	2032 (57.6)	725 (62.8)	.002	26.3	2137 (44.0)	193 (62.7)	<.001	8.3
Private	2150 (25.7)	377 (25.8)	.912	14.9	885 (25.1)	301 (26.1)	.499	25.4	1265 (26.1)	76 (24.7)	.591	5.7
No health coverage in addition to access to IHS services	2562 (30.6)	321 (22.0)	<.001	11.1	926 (26.2)	249 (21.6)	.001	21.2	1636 (33.7)	72 (23.4)	<.001	4.2
*Health status*												
Type of CVD												
Congestive heart failure	842 (10.0)	306 (20.9)	<.001	26.7	511 (14.5)	244 (21.1)	<.001	32.3	331 (6.8)	62 (20.1)	<.001	15.8
Ischemia	3666 (43.7)	669 (45.8)	.151	15.4	1847 (52.4)	552 (47.8)	.008	23.0	1819 (37.5)	117 (38.0)	.857	6.0
Other heart conditions^b^	3814 (45.5)	874 (59.8)	<.001	18.6	1513 (42.9)	648 (56.2)	<.001	30.0	2301 (47.4)	226 (73.4)	<.001	8.9
Cerebrovascular disease	1366 (16.3)	208 (14.2)	.046	13.2	534 (15.1)	166 (14.4)	.534	23.7	832 (17.1)	42 (13.6)	.112	4.8
Vascular disease	1941 (23.2)	375 (25.6)	.038	16.2	886 (25.1)	298 (25.8)	.63	25.2	1055 (21.7)	77 (25.0)	.179	6.8
Diabetes	3528 (42.1)	1154 (78.9)	<.001	24.6								
Renal disease	1236 (14.7)	341 (23.3)	<.001	21.6	771 (21.9)	313 (27.1)	<.001	28.9	465 (9.6)	28 (9.1)	.777	5.7
Amputation	113 (1.3)	20 (1.4)	.952	15.0	91 (2.6)	20 (1.7)	.101	18.0	22 (0.5)	0 (0.0)	.236	0.0
Neuropathy	1476 (17.6)	374 (25.6)	<.001	20.2	996 (28.2)	352 (30.5)	.139	26.1	480 (9.9)	22 (7.1)	.115	4.4
Mental health disorders	3028 (36.1)	514 (35.2)	.477	14.5	1254 (35.5)	421 (36.5)	.564	25.1	1774 (36.5)	93 (30.2)	.024	5.0
Alcohol/ drug use disorders	486 (5.8)	63 (4.3)	.022	11.5	159 (4.5)	44 (3.8)	.315	21.7	327 (6.7)	19 (6.2)	.699	5.5
Tobacco use disorders	1679 (20.0)	241 (16.5)	.002	12.6	615 (17.4)	180 (15.6)	.15	22.6	1064 (21.9)	61 (19.8)	.383	5.4
Liver disease	327 (3.9)	76 (5.2)	.021	18.9	158 (4.5)	64 (5.5)	.139	28.8	169 (3.5)	12 (3.9)	.701	6.6
*Clinical measures*												
Systolic blood pressure			<.001				<.001				.078	
<140 mm Hg	5768 (68.8)	1075 (73.5)		15.7	2284 (64.7)	839 (72.7)		26.9	3484 (71.8)	236 (76.6)		6.3
≥140 mm Hg	2332 (27.8)	369 (25.2)		13.7	1158 (32.8)	303 (26.3)		20.7	1174 (24.2)	66 (21.4)		5.3
Missing	282 (3.4)	18 (1.2)		6.0	86 (2.4)	12 (1.0)		12.2	196 (4.0)	6 (1.9)		3.0
LDL Cholesterol			<.001				<.001				<.001	
<100 mg/dL	3109 (37.1)	792 (54.2)		20.3	1684 (47.7)	664 (57.5)		28.3	1425 (29.4)	128 (41.6)		8.2
≥100 mg/dL	1976 (23.6)	332 (22.7)		14.4	832 (23.6)	245 (21.2)		22.7	1144 (23.6)	87 (28.2)		7.1
Missing	3297 (39.3)	338 (23.1)		9.3	1012 (28.7)	245 (21.2)		19.5	2285 (47.1)	93 (30.2)		3.9
A1c							<.001					
<8%					1978 (56.1)	740 (64.1)		27.2				
≥8%					921 (26.1)	347 (30.1)		27.4				
Missing					629 (17.8)	67 (5.8)		9.6				
*Medications*												
Medications dispensed												
Q1: ≤27	2259 (27.0)	231 (15.8)	<.001	9.3	513 (14.5)	181 (15.7)	<.001	26.1	1746 (36.0)	50 (16.2)	<.001	2.8
Q2: 28‐61	2155 (25.7)	253 (17.3)		10.5	687 (19.5)	148 (12.8)		17.7	1468 (30.2)	105 (34.1)		6.7
Q3: 62‐100	2076 (24.8)	407 (27.8)		16.4	1056 (29.9)	315 (27.3)		23.0	1020 (21.0)	92 (29.9)		8.3
Q4: >100	1892 (22.6)	571 (39.1)		23.2	1272 (36.1)	510 (44.2)		28.6	620 (12.8)	61 (19.8)		9.0
Anticoagulation medication use	344 (4.1)	414 (28.3)	<.001	55.2	144 (4.1)	219 (19.0)	<.001	60.3	200 (4.1)	195 (63.3)	<.001	49.4
*County and community measures*												
Household income			.072				.0825				.0089	
Lower poverty	4115 (49.1)	878 (60.1)		17.6	1646 (46.7)	678 (58.8)		29.2	2469 (50.9)	200 (64.9)		7.5
Higher poverty	4267 (50.9)	584 (39.9)		12.0	1882 (53.3)	476 (41.2)		20.2	2385 (49.1)	108 (35.1)		3.7
Educational attainment			.7835				.9875				.8587	
Higher attainment	3678 (43.9)	659 (45.1)		15.2	1568 (44.4)	513 (44.5)		24.7	2110 (43.5)	146 (47.4)		6.5
Lower attainment	4704 (56.1)	803 (54.9)		14.6	1960 (55.6)	641 (55.5)		24.6	2744 (56.5)	162 (52.6)		5.6

	Mean (SD)	Mean (SD)			Mean (SD)	Mean (SD)			Mean (SD)	Mean (SD)		
Travel time (minutes)	39.1 (27.5)	31.7 (22.5)	<.001		39.7 (28.9)	31.6 (22.8)	<.001		38.6 (26.5)	31.7 (21.4)	.0003	

Abbreviations: IHS, Indian Health Service, Q1 to Q4: quartiles 1 to 4.

a Characteristics are for the baseline year, fiscal year (FY) 2011, except for the county and community measures. The county measures for household income and educational attainment were derived from 2010‐2014 American Community Survey data. Community travel time estimates are for FY2012.

^b^ Other heart conditions include heart valve and pericardial conditions, congenital heart conditions, cardiac arrhythmias, and other heart conditions.

Use of clinical pharmacy services varied by type of CVD and comorbidity status. Over 25% of patients with congestive heart failure (CHF) used clinical pharmacy services. Patients with other heart conditions, excluding ischemia, and with vascular disease also had significantly higher use than patients without these conditions. Approximately 25% of adults with diabetes used clinical pharmacy services. Service use was significantly lower among adults with no SBP or cholesterol test values in FY2011.

Clinical pharmacy utilization was significantly associated with the level of medication use (*P* <.001) and use of anticoagulation medications (*P* <.001). Over half of adults dispensed anticoagulation medications used clinical pharmacy services.

There was no significant association between county‐level educational attainment and clinical pharmacy use. Although utilization was lower among adults who lived in counties with higher poverty, compared to adults who lived in lower poverty counties (12.0% vs 17.6%), this difference was not significant (*P* = .072). Clinical pharmacy users, compared to nonusers, had shorter travel times to services (31.7 vs 39.1 minutes, *P* <.001).

The results from the multivariable logistic regression analysis indicate that neither age nor gender were associated with clinical pharmacy utilization (Table 2). Adults with Medicaid coverage, compared to adults without Medicaid, had lower odds of service use (OR = 0.72; CI: 0.56‐0.93, *P* <.05).

**TABLE 2:** Multivariable logistic regression results predicting use of clinical pharmacy services in fiscal year 2012 among adults with cardiovascular disease (CVD). Five geographic locations

	Health status								
	CVD			CVD and diabetes			CVD absent diabetes^a^		
Characteristics	OR	CI	*P*‐value	OR	CI	*P*‐value	OR	CI	*P*‐value
*Demographic*									
Age (years)			.7537			.7052			.7432
18‐35	0.77	(0.46,1.3)		1.01	(0.49,2.07)		0.65	(0.26,1.61)	
35‐45	0.82	(0.56,1.19)		0.76	(0.48,1.21)		0.98	(0.47,2.03)	
45‐55	0.90	(0.69,1.19)		0.83	(0.61,1.14)		1.08	(0.58,2.02)	
55‐65	0.88	(0.71,1.11)		0.87	(0.67,1.12)		0.88	(0.52,1.48)	
65+									
Sex			.1545			.4747			.1688
Male	1.11	(0.96,1.28)		1.06	(0.9,1.26)		1.24	(0.91,1.68)	
*Health coverage*									
Medicaid	0.72	(0.56,0.93)	.0125	0.70	(0.52,0.94)	.0171	1.04	(0.6,1.81)	.8875
Medicare	1.03	(0.83,1.29)	.7712	0.95	(0.74,1.23)	.714	1.40	(0.83,2.36)	.2089
Private insurance	1.10	(0.94,1.29)	.2441	1.18	(0.97,1.42)	.0912	0.98	(0.69,1.37)	.8845
*Health status*									
Type of CVD									
Congestive heart failure	1.22	(1.02,1.47)	.0346	1.13	(0.91,1.4)	.2668	1.54	(1.01,2.36)	.0451
Ischemia	0.95	(0.81,1.1)	.468	0.90	(0.76,1.08)	.2533	1.38	(0.98,1.93)	.0655
Other heart conditions^b^	1.40	(1.18,1.65)	<.0001	1.27	(1.05,1.54)	.0154	2.16	(1.51,3.1)	<.0001
Cerebrovascular disease	0.95	(0.77,1.16)	.5908	0.97	(0.76,1.22)	.7653	1.00	(0.65,1.53)	.9882
Vascular disease	1.24	(1.04,1.46)	.014	1.20	(0.99,1.47)	.0673	1.52	(1.06,2.18)	.0229
Diabetes	4.05	(3.28,4.99)	<.0001						
Renal disease	1.17	(0.98,1.4)	.0917	1.25	(1.02,1.53)	.0303	0.66	(0.39,1.12)	.1264
Amputation	0.42	(0.24,0.74)	.0028	0.44	(0.25,0.79)	.0061			
Neuropathy	1.26	(1.07,1.49)	.007	1.37	(1.13,1.64)	.001	0.87	(0.51,1.51)	.6247
Mental health disorders	0.98	(0.85,1.14)	.8111	1.02	(0.86,1.22)	.8028	0.87	(0.62,1.21)	.3978
Alcohol/drug use disorders	0.81	(0.57,1.14)	.2194	0.80	(0.53,1.2)	.2786	0.89	(0.46,1.7)	.7212
Tobacco use disorders	1.09	(0.9,1.32)	.367	1.01	(0.8,1.27)	.9251	1.19	(0.8,1.75)	.39
Liver disease	1.11	(0.81,1.53)	.5237	1.08	(0.75,1.56)	.6629	1.15	(0.54,2.44)	.7159
*Clinical measures*									
Systolic blood pressure			.0366			.2637			.7551
<140 mm Hg									
≥140 mm Hg	0.94	(0.81,1.1)		0.93	(0.77,1.11)		1.01	(0.71,1.44)	
Missing	0.49	(0.28,0.86)		0.58	(0.28,1.2)		1.44	(0.56,3.72)	
LDL cholesterol			.001			.1686			.0276
<100 mg/dL									
≥100 mg/dL	0.88	(0.74,1.05)		0.93	(0.77,1.11)		1.07	(0.74,1.55)	
Missing	0.67	(0.55,0.83)		0.87	(0.66,1.15)		0.65	(0.45,0.95)	
A1c						<.0001			
<8%									
≥8%				1.14	(0.94,1.37)				
Missing				0.41	(0.29,0.59)				
Medications									
Medication use quartile			.0701			.0061			.8052
Q1: ≤27									
Q2: 28‐61	1.01	(0.75,1.34)		0.94	(0.64,1.39)		1.05	(0.67,1.65)	
Q3: 62‐100	1.21	(0.91,1.61)		1.32	(0.9,1.92)		0.96	(0.59,1.56)	
Q4: >100	1.32	(0.98,1.77)		1.48	(1.01,2.17)		0.84	(0.48,1.47)	
Anticoagulation medication use	20.85	(16.74,25.96)	<.0001	8.91	(6.62,11.98)	<.0001	39.66	(27.89,56.4)	<.0001
*County and community measures*									
Higher poverty counties	0.50	(0.24,1.01)	.0535	0.57	(0.25,1.29)	.1754			
Lower educational attainment counties	0.84	(0.45,1.55)	.5753	0.88	(0.42,1.84)	.7319			
Travel time (10 minute increases)	0.87	(0.83,0.92)	<.0001	0.86	(0.81,0.92)	<.0001	0.85	(0.77,0.93)	.0005
*Interaction*			<.0001						
Anticoagulation medication use among adults with diabetes	6.78	(5.15,8.91)							
Anticoagulation medication use among adults without diabetes	64.14	(46.8,87.91)							

*Note*: The *P*‐value was determined from Type 3 tests for fixed effects.

Abbreviations: CI, confidence interval; OR, odds ratio; Q1 to Q4, quartiles 1 to 4.

^a^ Due to small numbers, we estimated the regression twice. This table includes results for the regression with the travel time measure and community random effects. In the second regression, which included county income and education measures, excluded the travel time measure, and included county random effects, adults who lived in higher poverty counties had significantly lower odds of clinical pharmacy use [OR = 0.39, (0.21,0.73)].

b Other heart conditions include heart valve and pericardial conditions, congenital heart conditions, cardiac arrhythmias, and other heart conditions.

The estimated ORs for utilization for adults with CHF, other types of heart disease, and vascular disease were 1.22 (CI: 1.02‐1.47, *P* <.05), 1.40 (CI: 1.18‐1.65, *P* <.001), and 1.24 (CI: 1.04‐1.46, *P* <.05), respectively. Diabetes and anticoagulation medication use were associated with substantially higher odds of utilization (OR = 4.05: CI: 3.28‐4.99, *P* <01; OR = 20.85; CI: 16.74‐25.96, *P* <.001; respectively). No SBP and no cholesterol test values were associated with significantly lower odds of service use (OR = 0.49: CI: 0.28‐0.86, *P* <.05; OR = 0.67, CI: 0.55‐0.83, *P* <.001; respectively).

While the odds of clinical pharmacy utilization among adults who lived in higher poverty counties were 50% (CI: 0.24‐1.01) lower than those for adults who lived in lower poverty counties, the association was only marginally significant (*P* = .0535). Adults who lived in communities with longer travel times to services had lower odds of service use. For each 10‐minute increase in travel time, the odds of using services decreased by 13% (OR = 0.87; CI: 0.83‐0.92, *P* <.05).

According to multivariable logistic regression results for adults with CVD and diabetes, having a heart condition other than CHF or ischemia was associated with higher odds of service use (OR = 1.27; CI: 1.05‐1.54, *P* <.05). The odds of using services were lower among those who had amputation‐related services (OR = 0.44; CI: 0.25‐0.79, *P* <.001) and higher among those with neuropathy (OR = 1.37; CI: 1.13‐1.64, *P* <.01) and those with renal disease (OR = 1.25, CI: 1.02‐1.53, *P* <.05). Not having an HbA1c test value was associated with lower odds of service use, compared to having an HbA1c <8% (OR = 0.41; CI: 0.29‐0.59, *P* <.001).

Among adults with CVD absent diabetes, adults with CHF, a heart condition other than CHF or ischemia, or vascular disease had higher odds of service use. Adults without a cholesterol test value and those living in higher poverty counties had lower odds of service use.

Clinical pharmacy use varied by diabetes status among patients who used anticoagulation medications. Sixty percent of adults with CVD and diabetes used clinical pharmacy services compared to 49.4% of adults with CVD absent diabetes. The multivariable regression analysis results confirmed the statistical significance of this finding (*P* <.0001).

### 3.3 SBP associated with clinical pharmacy utilization

Among adults with CVD, the percentage with elevated SBP in FY2013 was lower among clinical pharmacy users than nonusers (22.7% vs 28.0%, *P* <.001). Using the propensity score matching method, the odds of having elevated SBP in FY2013 for clinical pharmacy users were 29% lower (OR = 0.71; CI: 0.58‐0.87, *P* <.001) than nonusers. This finding was consistent with the result from the Disease Risk Score model (OR = 0.74, CI: 0.61‐0.91, *P* <.01; see Table S3).

## 4 DISCUSSION

This is the first study, to our knowledge, identifying patient characteristics associated with clinical pharmacy utilization among AI/ANs with CVD who access services through IHS and Tribal health programs. Four key findings from this utilization study, summarized below, may collectively inform efforts to assess provider referrals to and the availability of clinical pharmacy services; improve patient knowledge of clinical pharmacy goals and outcomes; enhance patient outreach via telephone, text, email, and other methods; and increase care coordination among patient medical home team members^48^ via office and electronic health systems, thereby facilitating higher clinical pharmacy utilization by patients referred to them.

First, due to medication treatment needs, we anticipated adults with CHF, other types of heart conditions excluding ischemia, or vascular disease would use services more than their counterparts without these conditions, which was confirmed in our results. Due to medical monitoring and complex dosing of anticoagulants, we expected most adults who used anticoagulants to use clinical pharmacy services. In this study, just over half of adults taking anticoagulation medications used these services.

Second, the odds of service utilization were four times higher among adults with CVD and diabetes, compared to adults with CVD absent diabetes. Additionally, clinical pharmacy service utilization by adults treated for coagulopathy was significantly higher among patients with both CVD and diabetes compared to CVD patients who did not have diabetes (utilization rates of 60.3% and 49.4%, respectively). While this may be due to differences in their clinical needs, additional factors may influence their clinical pharmacy utilization (eg, other health care providers emphasizing medication management among patients with both CVD and diabetes). These findings suggest opportunities to enhance outreach and care coordination for adults with CVD who do not have a concurrent diagnosis of diabetes.

Third, patients who experience challenges managing chronic conditions and/or accessing health services, in general, may in turn experience challenges accessing clinical pharmacy services. Adults with CVD who did not have a cholesterol test in FY2011 and adults with CVD and diabetes who were classified as having had an amputation‐related procedure were less likely to use clinical pharmacy services.

Fourth, we found 3 of 4 SDOH, referenced in the Healthy People SDOH framework,^11^, ^41^ were associated with lower utilization. Adults with Medicaid coverage, which generally indicates low household income, were 72% less likely to use services than adults without Medicaid coverage. Clinical pharmacy utilization among adults who lived in higher poverty counties was half that of adults who lived in counties with lower poverty; although, this result was only marginally significant. With respect to travel time, each 10‐minute increase in patient travel time to services reduced the patient's odds of using services by 13%. Increased outreach and care coordination, with a stronger focus on patient transportation and other barriers, may, upon referral, ultimately increase patients' ability to access services.

Finally, with regard to our secondary study objective, clinical pharmacy use was associated with improvements in SBP. Adults with CVD who used clinical pharmacy services in FY2012, compared to nonusers, were 29% less likely to have elevated SBP in FY2013. This blood pressure analysis expanded upon that described in a 2019 study of clinical pharmacy services provided within federally funded facilities, which included I/T facilities.^19^ In that study, patients with hypertension who were seen by clinical pharmacists had a mean decrease in SBP of 11.2 mm Hg, from an average baseline value of 144 mm Hg.

There are limitations to this study. First, the data are from FY2011‐2013. Despite the age of the data, the results fill in important knowledge gaps and make relevant contributions to the literature. We described clinical pharmacy service utilization among a low‐income, largely rural AI/AN population that has a high chronic disease burden but is severely understudied. Better understanding of the provision of clinical pharmacy services within I/T hospitals and clinics promises to fill this knowledge gap. Also, the study addresses a crucial time when clinical pharmacy services expanded within I/T hospitals and clinics. Our findings about the relationships among patient health status, SDOH, and clinical pharmacy use may inform enhancements to clinical pharmacy referrals, utilization, and service availability and may be reaffirmed in future studies.

Second, we could not examine the distinction between pharmacists who acted as consultants or PCPs due to data limitations,^19^ and the analysis included only 1 year of clinical pharmacy utilization and one outcome. Next steps are to obtain data for recent time periods and examine clinical pharmacy utilization by type and level of services provided, utilization across multiple years, and a broader array of health outcomes.

Other limitations merit consideration. The health status measures were derived from I/T and PRC electronic data; many diagnostic codes recorded in non‐I/T provider medical records for services not paid for by the PRC program were excluded. This may have negatively biased the prevalence of reported chronic conditions. In addition, we did not include clinical pharmacy utilization at non‐IT facilities, where we hypothesize such utilization may be limited except in Veterans Administration health facilities through which a small percentage of AI/ANs included in this study may have accessed such services. Lastly, our results are generalizable to the AI/ANs who lived in the geographic areas studied; these findings do not reflect clinical pharmacy utilization of AI/AN peoples who live elsewhere or who do not utilize I/T services.

## 5 CONCLUSIONS

Despite these limitations, our findings improve knowledge of I/T clinical pharmacy service utilization by patient health status and SDOH. Study results indicate that some AI/AN adults with CVD who may benefit from clinical pharmacy utilization may not access such services due to barriers related to travel time, household income, or other factors influencing service use. Increased patient knowledge of these services and patient care coordination may facilitate their use of clinical pharmacy services and, ultimately, optimize their care and improve health outcomes. The COVID‐19 pandemic greatly impacted access to care through the provision of telehealth services. The provision of telephonic, virtual, and other types of telehealth visits has likely increased access to clinical pharmacy services for many patients and merits future study.

Between 2012 and 2017, I/T hospitals and clinics increased the provision of pharmacist‐provided services. In 2017, the NCPS Committee required that pharmacists practice using a more comprehensive approach to providing care, as compared to focusing on specific health needs. Accordingly, the NCPS Committee provides national certification for clinical pharmacists for an advanced scope of practice aimed at comprehensive patient management (also known as CMM). Within I/T facilities clinical pharmacists typically provide comprehensive patient management as a member of the patients' medical home team^49^; the provision of clinical pharmacy services in this manner is associated^24^ with improved patient outcomes and resource savings. As of July 2021, there were 115 pharmacists nationally certified to provide Comprehensive Clinical Pharmacy Services.^50^ Clinical pharmacists may also be credentialed locally, proving comprehensive care. Results from this study may inform assessments of clinical pharmacy referrals, utilization, and service availability as the provision of comprehensive clinical pharmacy services increases.

## CONFLICT OF INTEREST

The authors declare no potential conflict of interest.

## Supporting information


**Data S1** Supporting Information
