# Supplementary material for: Use of clinical pharmacy services by American Indians and Alaska Native adults with cardiovascular disease
Source: J Am Coll Clin Pharm. 2022 May 25;5(8):800–11. doi: 10.1002/jac5.1651 (PMC9544095; doi:10.1002/jac5.1651)
Supplement: Supplementary file 1 — Data S1 Supporting Information [file JAC5-5-800-s001.docx]

**Supplemental Materials**

**Use of Clinical Pharmacy Services by**

**American Indians and Alaska Native adults with Cardiovascular Disease**

This document provides additional information on 1) the characteristics of the American Indian and Alaska Native (AI/AN) adults with cardiovascular disease (CVD) included in the study population, 2) their use of clinical pharmacy services, 3) statistical methods employed to estimate the association of elevated systolic blood pressure (SBP) in FY2013 with FY2012 clinical pharmacy utilization and 4) the results of those statistical analyses.

1. Characteristics of the study population

Table S1 provides information on characteristics of the AI/AN adults with CVD included in the study population for FY2011. Data are provided for all adults with CVD and for adults with CVD stratified by diabetes status. These data supplement those provided in Table 1.

1. Clinical pharmacy utilization during FY2022 by AI/AN adults with CVD

Table S2 provides information on clinical pharmacy utilization during FY2022 by AI/AN adults with CVD. Data are provided for all adults with CVD and for adults with CVD stratified by diabetes status. Clinical pharmacy utilization measures include the percentage of adults who had one or more clinical pharmacy visits during 2012 among all adults and the average number of clinical pharmacy visits the clinical pharmacy users had during 2012.

1. Statistical methods employed to estimate the association of elevated SBP in FY2013 with FY2012 clinical pharmacy utilization

For the secondary study goal, we employed a propensity score matched conditional logistic regression and multivariable logistic regression to estimate the association between clinical pharmacy use in FY2012 with elevated SBP in FY2013 among adults with CVD.^[[1]](#footnote-1)^^[[2]](#footnote-2)^ This statistical approach was selected to control for potential bias due to patient self-selection into use or non-use of clinical pharmacy services in this observational study. We conducted a sensitivity analysis by estimating the same relationship using an alternative statistical method developed to control for selection bias, the Disease Risk Score model.^[[3]](#footnote-3)^ These analyses only included data for 4 of the 5 geographic areas due to SBP data quality issues in one geographic area.

- 1. Propensity Score Model

To address nonrandom assignment of the AI/AN adults into 2 groups (i.e., clinical pharmacy users and non-users in FY2012), we employed propensity score matching. Equation 1 of the model estimated a patient’s propensity to use clinical pharmacy services during FY2012, based on baseline (i.e., FY2011) patient characteristics that included age, sex, health coverage, health status (conditions and clinical values), medication use, measures of social determinants of health (SDOH), and project site (See Table S1). The clinical values included FY2011 SBP. Equation 2 compared SBP during FY2013 between FY2012 users and non-users, using a 1:1 matched sample of users and non-users who were matched by propensity scores. Equation 2 was an unadjusted conditional logistic regression.

- 1. Disease Risk Score Model

The odds ratio for the Disease Risk Score method was estimated using 4 steps. First, we estimated a multiple logistic regression equation predicting high SBP among clinical pharmacy non-users to estimate their predicted probability of high SBP (i.e., predicted probabilities or disease risk score). SBP was estimated as a function of age, sex, health coverage, health status (conditions and clinical values), medication use, and project site. The clinical values included FY2011 SBP. Second, we estimated the disease risk scores of clinical pharmacy users from the users’ patient characteristics and the coefficients of the regression estimated in Step 1. Third, we created a 1:1 matched sample of users and non-users, based on the disease risk scores of the users and non-users. Fourth, we estimated an unadjusted conditional logistic regression to assess the relationship between clinical pharmacy use and SBP. We evaluated the risk scores using the “dry-run” analysis.^3^

1. The relationship between FY2012 clinical pharmacy utilization with FY2013 elevated SBP

Table S.3 provides results for the analyses described in Section 3.

| **Table S1. Characteristics of American Indian and Alaska Native adults with cardiovascular disease (CVD). Five geographic locations. Fiscal year 2011.*** | | | | | | |
| --- | --- | --- | --- | --- | --- | --- |
|  |  | **Health Status** | | | | |
|  |  | **CVD** |  | **CVD and diabetes** |  | **CVD absent diabetes** |
| **Characteristics** |  | **N (%)** |  | **N (%)** |  | **N (%)** |
| All adults |  | 9,844 (100.0) |  | 4,682 (100.0) |  | 5,162 (100.0) |
|  |  |  |  |  |  |  |
| **Demographic** |  |  |  |  |  |  |
| Age |  |  |  |  |  |  |
| 18-35 |  | 566 (5.8) |  | 68 (1.5) |  | 498 (9.7) |
| 35-45 |  | 753 (7.6) |  | 226 (4.83) |  | 527 (10.2) |
| 45-55 |  | 1,822 (18.5) |  | 797 (17.0) |  | 1,025 (19.9) |
| 55-65 |  | 2,640 (26.8) |  | 1,420 (30.3) |  | 1,220 (23.6) |
| 65+ |  | 4,063 (41.3) |  | 2,171 (46.4) |  | 1,892 (36.7) |
| Sex |  |  |  |  |  |  |
| Male |  | 4,888 (49.7) |  | 2,434 (52.0) |  | 2,454 (47.5) |
| Female |  | 4,956 (50.3) |  | 2,248 (48.0) |  | 2,708 (52.5) |
|  |  |  |  |  |  |  |
| **Health coverage** |  |  |  |  |  |  |
| Medicaid |  | 941 (9.6) |  | 434 (9.3) |  | 507 (9.8) |
| Medicare |  | 5,087 (51.7) |  | 2,757 (58.9) |  | 2,330 (45.1) |
| Private |  | 2,527 (25.7) |  | 1,186 (25.3) |  | 1,341 (26.0) |
| No health coverage in addition to access to IHS services |  | 2,883 (29.3) |  | 1,175 (25.1) |  | 1,708 (33.1) |
|  |  |  |  |  |  |  |
| **Health status** |  |  |  |  |  |  |
| Type of CVD |  |  |  |  |  |  |
| Congestive health failure |  | 1,148 (11.7) |  | 755 (16.1) |  | 393 (7.6) |
| Ischemia |  | 4,335 (44.0) |  | 2,399 (51.2) |  | 1,936 (37.5) |
| Other heart conditions† |  | 4,688 (47.6) |  | 2,161 (46.2) |  | 2,527 (49.0) |
| Cerebrovascular disease |  | 1,574 (16.0) |  | 700 (15.0) |  | 874 (16.9) |
| Vascular |  | 2,316 (23.5) |  | 1,184 (25.3) |  | 1,132 (21.9) |
| Diabetes |  | 4,682 (47.6) |  |  |  |  |
| Renal disease |  | 1,577 (16.0) |  | 1,084 (23.2) |  | 493 (9.6) |
| Amputation |  | 133 (1.4) |  | 111 (2.4) |  | 22 (0.4) |
| Neuropathy |  | 1,850 (18.8) |  | 1,348 (28.8) |  | 502 (9.7) |
| Mental health disorders |  | 3,542 (36.0) |  | 1,675 (35.8) |  | 1,867 (36.2) |
| Alcohol/drug use disorders |  | 549 (5.6) |  | 203 (4.3) |  | 346 (6.7) |
| Tobacco use disorders |  | 1,920 (19.5) |  | 795 (17.0) |  | 1,125 (21.8) |
| Liver disease |  | 403 (4.1) |  | 222 (4.7) |  | 181 (3.5) |
|  |  |  |  |  |  |  |
| **Clinical measures** |  |  |  |  |  |  |
| Systolic blood pressure |  |  |  |  |  |  |
| <140 mmHg |  | 6,843 (69.5) |  | 3,123 (66.7) |  | 3,720 (72.1) |
| ≥140 mmHg |  | 2,701 (27.4) |  | 1,461 (31.2) |  | 1,240 (24.0) |
| Missing |  | 300 (3.0) |  | 98 (2.1) |  | 202 (3.9) |
|  |  |  |  |  |  |  |
|  |  |  |  |  |  |  |
| LDL Cholesterol |  |  |  |  |  |  |
| <100 mg/dL |  | 3,901 (39.6) |  | 2,348 (50.1) |  | 1,553 (30.1) |
| ≥100 mg/dL |  | 2,308 (23.4) |  | 1,077 (23.0) |  | 1,231 (23.8) |
| Missing |  | 3,635 (36.9) |  | 1,257 (26.8) |  | 2,378 (46.1) |
| A1c |  |  |  |  |  |  |
| <8% |  |  |  | 2,718 (58.1) |  |  |
| ≥8% |  |  |  | 1,268 (27.1) |  |  |
| Missing |  |  |  | 696 (14.9) |  |  |
|  |  |  |  |  |  |  |
| **Medications** |  |  |  |  |  |  |
| Number of medications dispensed |  |  |  |  |  |  |
| Q1: <27 |  | 2,490 (25.3) |  | 694 (14.8) |  | 1,796 (34.8) |
| Q2: 28-61 |  | 2,408 (24.5) |  | 835 (17.8) |  | 1,573 (30.5) |
| Q3: 62-100 |  | 2,483 (25.2) |  | 1,371 (29.3) |  | 1,112 (21.5) |
| Q4: > 100 |  | 2,463 (25.0) |  | 1,782 (38.1) |  | 681 (13.2) |
| Anticoagulation medication use |  | 758 (7.7) |  | 363 (7.8) |  | 395 (7.7) |
|  |  |  |  |  |  |  |
| **County and community measures** |  |  |  |  |  |  |
| Household income |  |  |  |  |  |  |
| Lower poverty |  | 4,993 (50.7) |  | 2,324 (49.6) |  | 2,669 (51.7) |
| Higher poverty |  | 4,851 (49.3) |  | 2,358 (50.4) |  | 2,493 (48.3) |
| Educational attainment |  |  |  |  |  |  |
| Higher attainment |  | 4,337 (44.1) |  | 2,081 (44.4) |  | 2,256 (43.7) |
| Lower attainment |  | 5,507 (55.9) |  | 2,601 (55.6) |  | 2,906 (56.3) |
|  |  |  |  |  |  |  |
|  |  | Mean (SD) |  | Mean (SD) |  | Mean (SD) |
| Travel time (minutes) |  | 38.0 (26.9) |  | 37.7 (27.7) |  | 38.2 (26.2) |
|  |  |  |  |  |  |  |
| * Patient characteristics are for fiscal year (FY) 2011 except for county level measures for household income and educational attainment derived from American Community Survey data for 2010-2014 and FY2012 estimates for community drive time to services.  † Other heart conditions include heart valve and pericardial conditions, congenital heart conditions, cardiac arrhythmias, and other heart conditions. | | | | | | |

| **Table S2. Utilization of clinical pharmacy (CP) services among American Indian and Alaska Native adults with cardiovascular disease (CVD). Five geographic locations. Fiscal year 2012.** | | | | | | | | |
| --- | --- | --- | --- | --- | --- | --- | --- | --- |
|  | **CVD** | |  | **CVD and diabetes** | |  | **CVD absent diabetes** | |
| **Characteristics** | **% CP User** | **Average number of CP visits among CP users** |  | **% CP User** | **Average number of CP visits among CP users** |  | **% CP User** | **Average number of CP visits among CP users** |
| All adults | 14.9 | 5.3 |  | 24.7 | 4.3 |  | 6.0 | 9.2 |
|  |  |  |  |  |  |  |  |  |
| **Demographic** |  |  |  |  |  |  |  |  |
| Age (years) |  |  |  |  |  |  |  |  |
| 18-35 | 4.4 | 5.5 |  | 22.1 | 2.5 |  | 2.0 | 10.1 |
| 35-45 | 9.0 | 5.1 |  | 19.9 | 3.3 |  | 4.4 | 8.6 |
| 45-55 | 11.6 | 5.0 |  | 20.6 | 3.9 |  | 4.7 | 8.7 |
| 55-65 | 15.2 | 4.5 |  | 24.0 | 3.8 |  | 5.0 | 8.3 |
| 65+ | 18.6 | 5.9 |  | 27.1 | 4.8 |  | 8.8 | 9.7 |
| Sex |  |  |  |  |  |  |  |  |
| Male | 16.0 | 5.4 |  | 24.7 | 4.3 |  | 7.4 | 9.3 |
| Female | 13.7 | 5.2 |  | 24.6 | 4.3 |  | 4.7 | 9.0 |
|  |  |  |  |  |  |  |  |  |

*Clinical pharmacy (CP) users were adults who had 1 or more clinical pharmacy visits during FY2012.

| **Table S3. Changes in high systolic blood pressure (SBP) between FY2011 and FY2013, among American Indian and Alaska Native adults with cardiovascular disease (CVD), associated with use of clinical pharmacy (CP) services in FY2012. Four geographic locations.** | | | | |
| --- | --- | --- | --- | --- |
|  |  | CP non-user | CP user | Significance |
| Section 1: Percent of adults with high SBP* |  | % high SBP | % high SBP |  |
|  |  |  |  |  |
| FY2011 |  | 28.8% | 25.6% | p<0.05 |
| FY2013 |  | 28.0% | 22.7% | p<0.001 |
|  |  |  |  |  |
| Section 2: Odds ratio associated with FY2012 CP use predicting high SBP in FY2013† |  | Odds ratio | 95% Confidence interval | Significance |
|  |  |  |  |  |
| Method: Propensity score |  | 0.71 | (0.58, 0.87) | p<0.001 |
| Method: Disease risk score |  | 0.74 | (0.61, 0.91) | p<0.01 |
|  |  |  |  |  |
| High SBP is defined as ≥140 mmHg.  * Section 1: Data for adults with missing SBP during FY2011 and FY2013 were excluded. In fiscal year (FY) 2011, 3.1% of adults were missing a SBP value, in FY2013 the percent was 21.5%. Significance was determined using Chi-Square tests among non-missing values.  † Section 2: Data for adults with missing SBP during FY2013 were excluded from Section 2. See Sections 3.1 and 3.2 of these Supplemental materials for information on methods used to estimate the odds ratios for the two methods (propensity score and Disease Risk Score).  Note: Due to the complexity of the two statistical models and the small number of patients with CVD without diabetes who used clinical pharmacy services, subgroup analyses for adults by diabetes status were not conducted. | | | | |

1. Guo SY, Fraser MW. *Propensity score analysis: Statistical methods and applications*. SAGE Publications, Inc.; 2009 [↑](#footnote-ref-1)
2. Medaglio D, Stephens-Shields AJ, and Leonard CE. Research and scholarly methods: Propensity scores*. J Am College Clin Pharmacy*. 2022; 5:467-475.<https://doi.org/10.1002/jac5.1591> [↑](#footnote-ref-2)
3. Wyss R, Hansen BB, Ellis AR, et al. The "Dry-Run" Analysis: A method for evaluating risk scores for confounding control. *Am J Epidemiol*. 2017;185(9):842-852. doi:10.1093/aje/kwx032 [↑](#footnote-ref-3)
